# Interplay of an Obesity-Based Genetic Risk Score with Dietary and Endocrine Factors on Insulin Resistance

**DOI:** 10.3390/nu12010033

**Published:** 2019-12-21

**Authors:** Omar Ramos-Lopez, José Ignacio Riezu-Boj, Fermin I. Milagro, Marta Cuervo, Leticia Goni, J. Alfredo Martinez

**Affiliations:** 1 Department of Nutrition, Food Science and Physiology, and Center for Nutrition Research, University of Navarra, 31008 Pamplona, Spain; oscar.omar.ramos.lopez@uabc.edu.mx (O.R.-L.); jiriezu@unav.es (J.I.R.-B.); fmilagro@unav.es (F.I.M.); mcuervo@unav.es (M.C.); lgoni@unav.es (L.G.); 2 Medicine and Psychology School, Autonomous University of Baja California, Tijuana 22427, Mexico; 3 Navarra Institute for Health Research (IdiSNA), 31008 Pamplona, Spain; 4 CIBERobn, Fisiopatología de la Obesidad y la Nutrición, Carlos III Health Institute, 28029 Madrid, Spain

**Keywords:** insulin resistance, obesity, genetic risk score, diet, metabolic factors, personalized nutrition

## Abstract

This study aimed to nutrigenetically screen gene-diet and gene-metabolic interactions influencing insulin resistance (IR) phenotypes. A total of 232 obese or overweight adults were categorized by IR status: non-IR (HOMA-IR (homeostatic model assessment - insulin resistance) index ≤ 2.5) and IR (HOMA-IR index > 2.5). A weighted genetic risk score (wGRS) was constructed using 95 single nucleotide polymorphisms related to energy homeostasis, which were genotyped by a next generation sequencing system. Body composition, the metabolic profile and lifestyle variables were evaluated, where individuals with IR showed worse metabolic outcomes. Overall, 16 obesity-predisposing genetic variants were associated with IR (*p* < 0.10 in the multivariate model). The wGRS strongly associated with the HOMA-IR index (adj. R squared = 0.2705, *p* < 0.0001). Moreover, the wGRS positively interacted with dietary intake of cholesterol (P int. = 0.002), and with serum concentrations of C-reactive protein (P int. = 0.008) regarding IR status, whereas a negative interaction was found regarding adiponectin blood levels (P int. = 0.006). In conclusion, this study suggests that interactions between an adiposity-based wGRS with nutritional and metabolic/endocrine features influence IR phenotypes, which could facilitate the prescription of personalized nutrition recommendations for precision prevention and management of IR and diabetes.

## 1. Introduction

Obesity usually has a negative metabolic impact associated to an excessive and abnormal adipose tissue deposition and function [1], which affects several physiological processes such as glucose homeostasis and insulin sensitivity [2].

Insulin resistance (IR) is characterized by complete or partial insufficiency of insulin action on target tissues, being one of the first physiological abnormalities that precede the development of type 2 diabetes (T2D) and being usually related to concomitant metabolic chronic diseases such as obesity, non-alcoholic fatty liver disease, and atherosclerosis [3]. In turn, this condition is associated with concomitant pathological processes including hyperlipidemia, ectopic lipid accumulation, low-grade inflammation, endoplasmic reticulum stress, and oxidative cell damage [4].

Given the high heterogeneity of chronic diseases, efforts have been made to stratify clinical/metabolic outcomes in order to personalize the treatment. In this context, novel subgroups of adult-onset diabetes with variable disease progression have been postulated [5]. Studies have shown that body fat accumulation in the android or gynoid compartments has been associated with different cardiometabolic risk factors, including IR [6]. Additionally, metabolically healthy and unhealthy phenotypes have emerged in subjects with excessive adiposity [7].

Although the precise molecular mechanisms involved in the onset and progression of IR are not completely understood, studies suggest that genetic and environmental factors may influence IR predisposition [8]. Accordingly, candidate, linkage and genome-wide association studies have allowed the identification of a number of genetic variants associated with IR and related traits [9]. Moreover, Westernized food patterns in conjunction with sedentary behaviors contribute to the increased risk of IR and T2D [10]. Furthermore, potential interactions between genetic polymorphisms and nutritional factors have been pioneerly proposed to modulate the risk of developing IR [11,12] as well as changes in IR status in response to dietary interventions may be gene-dependent [13,14].

Together, these findings highlight the importance to study the complex interactions between the genetic background and lifestyles underlying IR in order to translate this information into innovative strategies aimed to the early detection, accurate diagnosis, and effective clinical management of IR and T2D. The aim of this study was to nutrigenetically screen gene-diet and gene-metabolic interactions influencing IR phenotypes in overweight/obese subjects.

## 2. Materials and Methods

### 2.1. Participants

This cross-sectional study included 232 Caucasian non-consanguineous overweight (BMI: 25–29.9 kg/m^2^) or obese (BMI: 30–40 kg/m^2^) adults from the Obekit study. Major exclusion criteria were a clinical history of type 1 diabetes or cardiovascular disease; T2D patients treated with insulin; pregnant or lactating women; and use of medication that could affects body weight or lipid/glucose levels (antipsychotic and antidepressant drugs, or corticosteroids). The study protocol was approved by the Research Ethics Committee of the University of Navarra (ref. 132/2015). The characteristics of this research project including study design, and registration have been previously reported [15,16]. Approval by the Research Ethics Committee of the University of Navarra (ref. 132/2015) was obtained, and the ethical principles of the 2013 Helsinki Declaration concerning medical research involving human subjects were carefully followed. In addition, all participants signed out the written informed consent.

### 2.2. Study Variables

Demographic characteristics, anthropometric and body composition measurements, the metabolic/inflammatory profile, and the lifestyle (including dietary intake and the level of physical activity) at baseline as well as the genotype of 95 obesity-predisposing single nucleotide polymorphisms (SNPs) were retrieved from the Obekit database. Methodology details regarding anthropometric assessment, lifestyle data collection, and blood samples processing have been previously provided [15,16]. Additionally, the selection procedure and genomic information of the 95 genetic variants have been recently reported [17]. The homeostatic model assessment-insulin resistance (HOMA-IR (homeostatic model assessment - insulin resistance)) index was calculated according to the Matthews formula: fasting insulin (µU/L) x fasting glucose (nmol/L)/22.5 [18]. Subjects were categorized by IR status according to the HOMA-IR index: non-IR (NIR) (HOMA-IR index ≤ 2.5) and IR (HOMA-IR index > 2.5) following accepted criteria [19]. The triglyceride-glucose index (TyG index) was estimated as: TyG index = (ln(fasting triglycerides (mg/dL) × fasting plasma glucose (mg/dL)/2)), as described elsewhere [20].

### 2.3. GRS Calculation

First screening consisted of comparing the frequency of IR between genotypes of the 95 SNPs to select those with a *p* value < 0.20, which were adjusted by BMI, waist circumference, and body fat in order to rule out random baseline adiposity differences. Next, genotypes with similar non-significant effects were clustered in a single group and coded as risk and non-risk groups. A risk genotype was defined as that associated with a higher frequency of IR. Then, SNPs whose risk genotypes presented at least a marginal statistical trend (*p* < 0.10 in the multivariate model) were finally selected, excluding those with low sample (<10%) in either NIR and IR categories or due to collinearity. Using these SNPs, a weighted genetic risk score (wGRS) was computed by multiplying the risk genotypes at each locus for the corresponding effect sizes (β-coefficients) on IR, and then summing the products, assuming that all selected SNPs had independent effects and contributed in an additive manner to IR, as previously reported [21].

### 2.4. Statistical Analyses

The distribution (normality) of the study variables was assessed by the Kolmogorov-Smirnov test. All main variables including HOMA-IR, PCR, adiponectin, dietary cholesterol, simple carbohydrates, and fiber were normally distributed (*p* > 0.05). Continuous and categorical variables were expressed as means ± standard errors (SE) and as numbers and percentages, respectively. Comparisons of body composition, metabolic, and dietary intake markers by IR status (NIR and IR) were carried out using ANCOVA tests adjusted by age, sex, and BMI. The levels of these markers were compared by dividing the population into two genetic categories: low genetic risk score (LGRS) and high genetic risk score (HGRS) based on the median value of wGRS (4.850). Differences in the proportions of risk genotypes between NIR and IR groups were assessed using chi squared tests. Mean values of wGRS according to NIR and IR phenotypes were compared by Student’s t tests. Multiple linear regression analyses adjusted by age, sex, and BMI were performed to evaluate the association between the wGRS and the HOMA-IR index. Additionally, logistic regression tests adjusted by the same covariates were run to assess the risk of develop IR in genetically susceptible individuals. Multivariate general linear models were applied to test interactions between the wGRS with dietary and metabolic factors in relation to IR after adjustment for age, sex, and BMI. Statistical analyses were performed in the statistical program Stata 12 (StataCorp LLC, College Station, TX, USA; www.stata.com). Statistical significance was established at *p* < 0.05. Figure plots were created using the GraphPad Prism^®^ software, version 6.0C (La Jolla, CA, USA) and Stata 12. Hardy–Weinberg equilibrium (HWE) and the analysis of molecular variance (AMOVA) were estimated using the Arlequin software, version 3.0 [22].

### 2.5. Functional Network Analyses

In order to show the interactions between the genes where significant SNPs are present, a multiprotein network was performed using the STRING (https://string-db.org/) with a low confidence interaction score (0.150). Functions enriched in the network were revealed according to the UniProt database (https://www.uniprot.org).

## 3. Results

### 3.1. Characteristics of the Study Population by Insulin Resistance Status

Anthropometrics, clinical and metabolic characteristics of the study population categorized by IR are reported (Table 1). The prevalence of IR was 22.8% in this sample. Participants with IR were older, had greater adiposity and worst blood lipid profile as well as lower levels of adiponectin than their NIR counterparts. Compatible findings were found when comparing HGRS and LGRS individuals, where HGRS subjects showed more clinically adverse phenotypes (Table S1).

### 3.2. Nutritional Profile Categorized by Insulin Resistance Status

Concerning the nutritional profile, higher intakes of simple carbohydrates and dietary cholesterol as well as lower intakes of dietary fiber were found in IR individuals than those exhibiting a NIR phenotype (Table 2). No additional differences were found between LGRS and HGRS groups for other macronutrients (Table S2).

### 3.3. Association of Genetic Variants with Insulin Resistance

The 16 obesity-predisposing genetic variants that were marginally or statistically associated with IR (*p* < 0.10), were incorporated into the wGRS. The characteristics and genome/statistical coding of each of these SNPs are reported (Table 3). The distribution of genotypes of the 16 SNPs was concordant with the HWE principle. Additionally, the AMOVA analyses revealed no significant genetic differentiation within the sample (*p* > 0 05). The following SNPs showed best association with IR: rs1800544 (*ADRA2A*), rs7903146 (*TCF7L2*), rs2289487 (*PLIN1*), rs12255372 (*TCF7L2*), rs894160 (*PLIN1*), rs206936 (*NUDT3*), rs1799883 (*FABP2*), rs2734827 (*UCP3*), rs10838738 (*MTCH2*), rs519887 (*ABCB11*), and rs7799039 (*LEP*). Ten SNPs were excluded from the analyses by low n sample or collinearity: rs8179183 (*LEPR*), rs1516725 (*ETV5*), rs10938397 (*GNPDA2*), rs1685325 (*UCP3*), rs1558902 (*FTO*), rs17817449 (*FTO*), rs8050136 (*FTO*), rs3751812 (*FTO*), rs9939609 (*FTO*), rs17069904 (*TNFRSF11A*). The score of the wGRS ranged from 1.23 to 9.75.

### 3.4. Multiprotein Network and Functional Enrichment Analyses

The results showed a statistically significant protein-protein interaction (PPI) enrichment *p*-value of 5.71 × 10^−14^, indicating that the proteins are at least in some way biologically connected, as a group (Figure S1). Functional enrichment analyses revealed two functions enriched in the network: obesity (false discovery rate = 0.0011) and diabetes (false discovery rate = 0.0226).

### 3.5. Association of the Weighted Genetic Risk Score with Insulin Resistance

**Figure 1:**
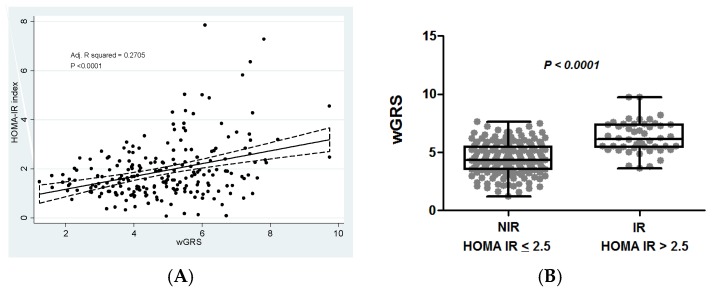
Associations between wGRS and insulin resistance. (**A**) Multiple linear regression evaluating the association between wGRS and HOMA-IR index adjusted by age, sex, and BMI. (**B**) Comparisons of wGRS mean values according to insulin resistance status. NIR: HOMA-IR ≤ 2.5; IR: HOMA-IR > 2.5; wGRS: weighted genetic risk score.

Associations between the wGRS and the HOMA-IR index are depicted (Figure 1). The wGRS positively associated with the HOMA-IR index (Figure 1A). Comparisons of wGRS mean values according to IR status are plotted (Figure 1). Statistically significant higher levels of wGRS were detected in the IR group (Figure 1B). Moreover, the HOMA-IR index increased 0.36 units by each unit of increase of the wGRS (B coefficient = 0.36, CI 95%, 0.26–0.46, *p* < 0.001). Additionally, HGRS individuals had 13.76 higher risk of develop IR than those carrying a LGRS (OR = 13.76, CI 95%, 5.18–36.54, *p* < 0.001).

### 3.6. Interactions between wGRS, Diet, and Metabolic Factors on Insulin Resistance

**Figure 2:**
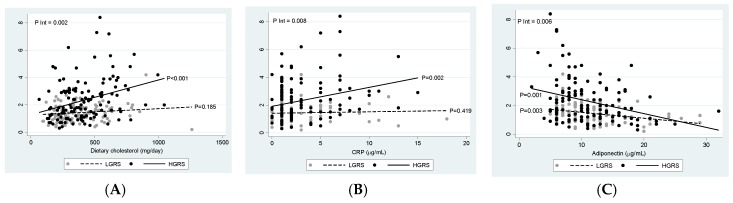
Estimations curves (95% CI) of gene-diet and gene-metabolic interactions concerning IR adjusted by age, sex, energy intake, and simple carbohydrates. (**A**) Interaction between the wGRS and dietary cholesterol in relation to the HOMA-IR index; (**B**) interaction between the wGRS and CRP concentrations respect to the HOMA-IR index; (**C**) interaction between the wGRS and adiponectin levels regarding the HOMA-IR index. LGRS: low genetic risk; HGRS: high-genetic risk; CRP: C-reactive protein; HOMA-IR index: homeostatic model assessment insulin resistance index.

Estimation curves of gene-diet and gene-metabolic interactions concerning IR are drawn (Figure 2). Interestingly, the wGRS positively interacted with dietary cholesterol to influence IR (Figure 2A). Thus, an increase in dietary cholesterol was associated with higher HOMA-IR index only in participants carrying a HGRS. Similar findings were raised regarding the blood concentrations of C-reactive protein (CRP) (Figure 2B). However, an opposite effect was detected in HGRS individuals in relation to serum adiponectin, where HOMA index decreased as adiponectin levels increased (Figure 2C).

## 4. Discussion

The prevalence of IR shows important variations across studies, mainly due to the use of different diagnosis tools and cut-off points as well as inherent differences in ethnicity, health status, and cultural issues [19]. In this investigation, IR was assessed via the HOMA-IR index because it is the most frequently used parameter in epidemiological studies [23], being considered by the international guidelines for the screening of high-risk groups [19]. According to this index, the 22.8% of the studied population had IR (HOMA IR > 2.5). Higher frequencies have been reported in Asians (51%) and Europeans (49%) using similar thresholds [24,25]. These findings highlight the heterogeneity of IR phenotypes among individuals and emphasize the importance of establishing more uniform criteria to define this metabolic feature as well as to investigate the role of the genetic makeup and the gene-diet interactions for precision management.

IR and hyperglycemia are two pathological conditions linked to obesity, aging, and chronic inflammation, which are major risk factors for the development of metabolic syndrome and T2D [26]. Accordingly, IR individuals in this study were older, heavier, and underwent a detrimental metabolic profile characterized by increased levels of blood lipids compared with normal NIR participants, being these outcomes similar to previous findings reported in the scientific literature [3,4].

Nutritional factors have been associated with IR and related inflammatory states in different populations [27,28]. In the studied population, participants with IR showed significantly higher consumptions of simple carbohydrates and dietary cholesterol and low intakes of dietary fiber than their NIR counterparts. In agreement with our results, a positive correlation between carbohydrate intake and HOMA-IR index was found in an Indian cohort [27]. Indeed, recent human studies support a dose response link between the consumption of simple carbohydrates and adverse metabolic changes, particularly when it is accompanied by an excessive intake of calories [29]. Nevertheless, findings from the Inter99 nonpharmacological intervention study revealed a lack of association of high content of carbohydrates, including simple sugars, with the probability of having IR [30]. Moreover, although dietary cholesterol has been identified as a major determinant of the severity of metabolic disorders (including IR) in animal models [31], current epidemiological and clinical evidences do not support consistently a relationship between dietary cholesterol and IR or T2D risks [32]. Regarding dietary fiber, large prospective cohort studies have consistently showed associations of a high-fiber intake with a reduction in the risk of developing IR and T2D [33]; however, these effects may vary by the types of fiber-rich foods and glycemic index, which were not analyzed in this study. Therefore, further investigation analyzing the role of diet in glucose homeostasis and insulin sensitivity is warranted.

Recent progress in genome sequencing techniques has added knowledge about the involvement of the genetic makeup in the susceptibility for developing IR [34]. In this study, 16 obesity-related genetic polymorphisms located at 13 genes were associated with IR. These genes participate in relevant physiological pathways such as lipid metabolism (*PLIN1*, *FABP2*, *ABCB11*, *NPC1*), energy balance (*LEP*, *UCP3*), presynaptic transmitter release (*ADRA2A*), cell signal transduction (*TCF7L2*, *NUDT3*), apoptosis (*AGTR2*), adipocyte differentiation (*MTCH2*), antioxidant response (*NFE2L3*), and amino acid processing (*MTHFR*) according to human gene database references (www.genecards.org). Also, multiprotein network analyses revealed partial, but significant interactions between these proteins. These results support a link between genetics, obesity and glucose homeostasis, as reviewed elsewhere [35,36].

Furthermore, in this research, a computed obesity-based wGRS using the IR-related alleles positively associated with the HOMA-IR index, which was independent of the adiposity degree, age, and sex. Additionally, higher levels of the wGRS were directly linked to IR. Moreover, individuals with a HGRS presented a more adverse metabolic profile compared with those carrying a LGRS. Instead, no differences in nutrient intakes between HGRS and LGRS groups were detected. According to our results, the genetic makeup is an important contributor to the pathogenesis of IR and related metabolic abnormalities. Consistently, an obesity GRS predicted the risk of IR in a Chinese children population [37]. Moreover, an adult-based GRS encompassing 53 SNPs was associated with the HOMA-IR index, metabolic syndrome traits, and altered fat distribution in a sample of Danish children and adolescents [38].

Complex gene-environment/lifestyle interactions modulate IR status and metabolic disease risk [39] and were also screened in this investigation. Thus, interactions between the wGRS with dietary intake of cholesterol and the serum levels of CRP and adiponectin in relation to IR were found. On the one hand, high CRP concentrations were associated with increased IR in genetically susceptible individual, supporting a link between low-grade chronic inflammation and IR [40]. On the other hand, high adiponectin levels were accompanied by reduced IR, indicating a protective role of adiponectin in relation to IR predisposition [41]. In addition, dietary cholesterol was positively associated with IR, especially in individuals at high-genetic risk. A similar study reported that the association between a GRS for glucose-stimulated insulin secretion and T2D risk was stronger in individuals with high energy intakes [42]. Similarly, the risk of T2D was exacerbated in subjects with high GRS for impaired insulin secretion capacity and consuming a low carbohydrate Western-style diet [43]. In addition, coffee drinkers (more than 10 cups coffee/week or 220 mg caffeine/day) were more susceptible to IR when carrying a high GRS for IR than those with a low GRS [44].

The strengths of this study include the screening of the interplay of an obesity-related wGRS with lifestyle data and metabolic/endocrine features influencing IR status in overweight/obese individuals. These analyses were adjusted by confounding factors affecting glucose homeostasis, such as age, sex, obesity degree, and total calorie intake [10]. Additionally, the possible effect of population stratification in our results was minimized due to the enrollment of a genetically homogeneous Caucasian/European population, as revealed by HWE and AMOVA analyses. On the other hand, the drawbacks of this research include a relatively small sample and the fact that the genetic findings of this investigation may not be generalizable to other ethnic groups exposed to different geographic/cultural environments. Therefore, further studies analyzing the association of these and other SNPs with IR and concomitant metabolic alterations in other populations are required in order to evaluate their applicability in a real-life scenario, although this research is a proof-of-principle.

Moreover, given the cross-sectional design of this study, no causality relationships between predictors and outcomes can be set. Furthermore, type I and type II errors cannot be completely ruled out despite the application of appropriate statistical settings. In addition, although the HOMA-IR index is a widely accepted indirect measurement of IR, the gold standard technique to assess this parameter could provide more information. Furthermore, interactions between the genetic makeup and other emerging factors affecting the host metabolism such as gut microbiota composition and epigenetic signatures also need to be explored [45].

Novel insights have emerged regarding the importance of categorizing disease outcomes with the aim of identifying high-risk individuals and customize treatments [5,6,7]. In this pioneer study, relevant differences in anthropometric, dietary, and clinical characteristics were evidenced in patients with overweight and obesity depending on the IR status, where interactions with the genetic background (wGRS) played a pivotal role. This information supports the heterogeneity of IR phenotypes between individuals and emphasizes the need for integrative individualized schemes of intervention for the control of obesity and related chronic diseases for precision nutrition [46].

## 5. Conclusions

In conclusion, this study suggests that interactions between the genetic makeup (exemplified in an obesity-based wGRS) and nutritional and metabolic features influence the IR phenotype, which could contribute to the understanding of IR pathogenesis as well as facilitate the prescription of personalized nutrition recommendations for the early prevention of T2D through the improvement of current therapeutic decision-making and the development of novel strategies using precision medicine approaches.

## Figures and Tables

**Table 1:** Anthropometric, clinical and biochemical characteristics of the study population categorized by insulin resistance status: NIR (*n* = 179), IR (*n* = 53).

Variable	NIR HOMA-IR ≤ 2.5	IR HOMA-IR > 2.5	*p*-Value
Age (y)	45.3 ± 0.7	48.6 ± 0.2	**0.038**
Sex (F/M)	129/50	32/21	0.105
Anthropometrics and clinical data			
Weight (kg)	84.9 ± 0.1	96.5 ± 0.4	**<0.001**
BMI (kg/m^2^)	30.9 ± 0.3	34.0 ± 0.4	**<0.001**
WC (cm)	101.2 ± 0.4	104.4 ± 0.8	**<0.001**
TFAT (kg)	36.4 ± 0.03	37.8 ± 0.05	**0.025**
VFAT (kg)	1.35 ± 0.03	1.73 ± 0.06	**0.052**
SBP (mmHg)	128 ± 1	129 ± 2	0.537
DBP (mmHg)	79 ± 1	81 ± 1	0.131
Biochemical profile			
Glucose (mg/dL)	93.8 ± 0.7	101.9 ± 1.4	**<0.001**
Insulin (mU/L)	5.9 ± 0.2	14.4 ± 0.4	**<0.001**
HOMA-IR index	1.40 ± 0.06	3.69 ± 0.12	**<0.001**
Total cholesterol (mg/dL)	217.4 ± 2.8	214.5 ± 5.4	0.649
LDL-c (mg/dL)	141.9 ± 2.5	137.9 ± 4.8	0.466
HDL-c (mg/dL)	57.1 ± 0.9	50.8 ± 1.7	**<0.001**
Triglycerides (mg/dL)	91.9 ± 3.6	129.3 ± 6.9	**<0.001**
TyG index (ratio)	8.29 ± 0.03	8.65 ± 0.06	**<0.001**
Uric acid (mg/dL)	5.13 ± 0.08	5.31 ± 0.16	0.336
ALT (IU/L)	22.3 ± 1.1	30.3 ± 2.0	**<0.001**
AST (IU/L)	21.6 ± 0.7	24.5 ± 1.4	0.064
Adiponectin (µg/mL)	11.9 ± 0.3	9.4 ± 0.6	**<0.001**
Leptin (ng/mL)	35.4 ± 1.6	41.1 ± 3.1	0.107
CRP (µg/mL)	2.50 ± 0.19	3.32 ± 0.38	0.065
TNFα (pg/mL)	0.98 ± 0.03	1.10 ± 0.06	0.082

Variables are expressed as means ± standard errors. NIR: non-insulin resistance; IR: insulin resistance; BMI: body mass index; WC: waist circumference; TFAT: total body fat; VFAT: visceral fat; SBP: systolic blood pressure; DBP: diastolic blood pressure; LDL-c: low-density lipoprotein cholesterol; HDL-c: high-density lipoprotein cholesterol; ALT: alanine aminotransferase; AST: aspartate aminotransferase; CRP: C-reactive protein; TNFα: tumoral necrosis factor alpha; TyG index: triglyceride-glucose index; HOMA-IR index: homeostatic model assessment insulin resistance index. Comparisons were performed using ANCOVA tests adjusted by age, sex, and BMI. Bold numbers indicate *p* < 0.05.

**Table 2:** Nutritional profile and physical activity patterns of the study subjects according to insulin resistance status.

Variable	NIR HOMA-IR ≤ 2.5	IR HOMA-IR > 2.5	*p*-Value
Energy (kilocalories/day)	1948 ± 38	2042 ± 72	0.265
Nutrient intake			
Complex carbohydrates (%E/day)	22.9 ± 0.5	24.2 ± 0.9	0.211
Simple carbohydrates (%E/day)	17.4 ± 0.8	19.9 ± 0.4	**0.011**
Total protein (%E/day)	19.5 ± 0.3	20.0 ± 0.6	0.496
Animal protein (%E/day)	13.4 ± 0.3	14.1 ± 0.6	0.305
Vegetal protein (%E/day)	5.5 ± 0.1	5.4 ± 0.2	0.490
Total fat (%E/day)	37.2 ± 0.5	37.6 ± 1.0	0.732
SFA (%E/day)	10.3 ± 0.2	10.4 ± 0.4	0.840
MUFA (%E/day)	15.7 ± 0.3	15.9 ± 0.5	0.791
PUFA (%E/day)	4.8 ± 0.1	4.9 ± 0.2	0.766
Dietary cholesterol (mg/day)	380 ± 14	457 ± 28	**0.017**
Fiber (g/day)	22.4 ± 0.6	19.6 ± 1.1	**0.031**
Water (mL/day)	1132 ± 24	1158 ± 47	0.633
Lifestyle factor			
Physical activity (METs/day)	24.2 ± 1.4	22.3 ± 2.7	0.555

Variables are expressed as means ± standard errors. Average intakes of macronutrients are adjusted by total energy consumption. SFA: saturated fatty acids; MUFA: monounsaturated fatty acids; PUFA: polyunsaturated fatty acids; METs: metabolic equivalents. Comparisons were performed by ANCOVA tests adjusted by age, sex, and BMI. Bold numbers indicate *p* < 0.05.

**Table 3:** Genomic and statistical characteristics of some selected SNPs associated with IR in overweight/obesity subjects.

No.	SNP ID	Gene	Alleles	Risk Genotype	Risk Genotype in NIR, *n* (%)	Risk Genotype in IR, *n* (%)	*p*-Value	HWE
1	rs1800544	*ADRA2A*	G/C	GG + CC	102 (57.0)	41 (77.4)	**0.007**	0.626
2	rs7903146	*TCF7L2*	C/T	CC	64 (35.8)	29 (54.7)	**0.013**	0.998
3	rs2289487	*PLIN1*	C/T	CC + CT	93 (52.0)	37 (71.2)	**0.014**	0.762
4	rs12255372	*TCF7L2*	G/T	GG	63 (35.2)	28 (52.8)	**0.021**	0.681
5	rs894160	*PLIN1*	C/T	CT + TT	80 (44.7)	33 (62.3)	**0.025**	0.996
6	rs206936	*NUDT3*	A/G	AA	98 (54.7)	38 (71.7)	**0.028**	0.450
7	rs1799883	*FABP2*	T/C	TT + TC	78 (43.6)	32 (60.4)	**0.031**	0.340
8	rs2734827	*UCP3*	G/A	GA + AA	92 (51.4)	36 (67.9)	**0.034**	0.935
9	rs10838738	*MTCH2*	A/G	AA	66 (36.9)	28 (52.8)	**0.038**	0.185
10	rs519887	*ABCB11*	T/C	TC + CC	115 (64.2)	42 (79.2)	**0.040**	0.288
11	rs7799039	*LEP*	G/A	GG	35 (19.7)	17 (33.3)	**0.040**	0.850
12	rs1055144	*NFE2L3*	C/T	CC + TT	121 (67.6)	43 (81.1)	0.057	0.344
13	rs1805081	*NPC1*	T/C	CC	22 (12.3)	12 (22.6)	0.061	0.311
14	rs11091046	*AGTR2*	A/C	CC	56 (32.9)	24 (47.1)	0.066	0.189
15	rs1801133	*MTHFR*	G/A	AA	20 (11.2)	11 (20.8)	0.072	0.397
16	rs1801131	*MTHFR*	T/G	TT	90 (50.3)	34 (64.2)	0.075	0.921

Data are expressed as number (percentage). Risk genotype comparisons were adjusted by BMI and WC. NIR: HOMA-IR ≤ 2.5; IR: HOMA-IR > 2.5. The SNPs are sorted in descending order based on the *p*-value. Comparisons of risk genotypes by IR categories were performed by Chi-square tests. HWE data are reported as *p* values of Chi-square tests. Bold numbers indicate *p* < 0.05. HWE: Hardy–Weinberg equilibrium.

## Supplementary Materials

The following are available online at https://www.mdpi.com/2072-6643/12/1/33/s1, Table S1: Anthropometric, clinical and biochemical characteristics of the study population categorized by insulin resistance genetic risk categories; Table S2: Nutritional profile and physical activity patterns of the study subjects according to insulin resistance genetic risk categories; Figure S1: Multiprotein network showing potential interactions between the 13 genes where SNPs are present. PPI enrichment *p*-value of 5.71 × 10^−14^.

## References

[1] Blüher M. (2009). Adipose tissue dysfunction in obesity. Exp. Clin. Endocrinol. Diabetes.

[2] Singla P., Bardoloi A., Parkash A.A. (2010). Metabolic effects of obesity: A review. World J. Diabetes.

[3] Samuel V.T., Shulman G.I. (2016). The pathogenesis of insulin resistance: Integrating signaling pathways and substrate flux. J. Clin. Investig..

[4] Schinner S., Scherbaum W.A., Bornstein S.R., Barthel A. (2005). Molecular mechanisms of insulin resistance. Diabet. Med..

[5] Ahlqvist E., Storm P., Käräjämäki A., Martinell M., Dorkhan M., Carlsson A., Vikman P., Prasad R.B., Aly D.M., Almgren P. (2018). Novel subgroups of adult-onset diabetes and their association with outcomes: A data-driven cluster analysis of six variables. Lancet Diabetes Endocrinol..

[6] Sari C.I., Eikelis N., Head G.A., Schlaich M., Meikle P., Lambert G., Lambert E. (2019). Android Fat Deposition and Its Association with Cardiovascular Risk Factors in Overweight Young Males. Front. Physiol..

[7] Ramos-Lopez O., Riezu-Boj J.I., Milagro F.I., Cuervo M., Goni L., Martinez J.A. (2019). Genetic and nongenetic factors explaining metabolically healthy and unhealthy phenotypes in participants with excessive adiposity: Relevance for personalized nutrition. Ther. Adv. Endocrinol. Metab..

[8] Højlund K. (2014). Metabolism and insulin signaling in common metabolic disorders and inherited insulin resistance. Dan. Med. J..

[9] Murea M., Ma L., Freedman B.I. (2012). Genetic and environmental factors associated with type 2 diabetes and diabetic vascular complications. Rev. Diabet. Stud..

[10] Kolb H., Martin S. (2017). Environmental/lifestyle factors in the pathogenesis and prevention of type 2 diabetes. BMC Med..

[11] Smith C.E., Arnett D.K., Corella D., Tsai M.Y., Lai C.Q., Parnell L.D., Lee Y.C., Ordovás J.M. (2012). Perilipin polymorphism interacts with saturated fat and carbohydrates to modulate insulin resistance. Nutr. Metab. Cardiovasc. Dis..

[12] Blanco-Rojo R., Delgado-Lista J., Lee Y.C., Lai C.Q., Perez-Martinez P., Rangel-Zuñiga O., Smith C.E., Hidalgo B., Alcala-Diaz J.F., Gomez-Delgado F. (2016). Interaction of an S100A9 gene variant with saturated fat and carbohydrates to modulate insulin resistance in 3 populations of different ancestries. Am. J. Clin. Nutr..

[13] Huang T., Huang J., Qi Q., Li Y., Bray G.A., Rood J., Sacks F.M., Qi L. (2015). PCSK7 genotype modifies effect of a weight-loss diet on 2-year changes of insulin resistance: The POUNDS LOST trial. Diabetes Care.

[14] Goni L., Qi L., Cuervo M., Milagro F.I., Saris W.H., MacDonald I.A., Langin D., Astrup A., Arner P., Oppert J.M. (2017). Effect of the interaction between diet composition and the PPM1K genetic variant on insulin resistance and β cell function markers during weight loss: Results from the Nutrient Gene Interactions in Human Obesity: Implications for dietary guidelines (NUGENOB) randomized trial. Am. J. Clin. Nutr..

[15] Goni L., Riezu-Boj J.I., Milagro F.I., Corrales F.J., Ortiz L., Cuervo M., Martínez J.A. (2018). Interaction between an ADCY3 Genetic Variant and Two Weight-Lowering Diets Affecting Body Fatness and Body Composition Outcomes Depending on Macronutrient Distribution: A Randomized Trial. Nutrients.

[16] Ramos-Lopez O., Riezu-Boj J.I., Milagro F.I., Goni L., Cuervo M., Martinez J.A. (2018). Differential lipid metabolism outcomes associated with ADRB2 gene polymorphisms in response to two dietary interventions in overweight/obese subjects. Nutr. Metab. Cardiovasc. Dis..

[17] Ramos-Lopez O., Riezu-Boj J.I., Milagro F.I., Cuervo M., Goni L., Martinez J.A. (2018). Prediction of Blood Lipid Phenotypes Using Obesity-Related Genetic Polymorphisms and Lifestyle Data in Subjects with Excessive Body Weight. Int. J. Genom..

[18] Matthews D.R., Hosker J.P., Rudenski A.S., Naylor B.A., Treacher D.F., Turner R.C. (1985). Homeostasis model assessment: Insulin resistance and beta-cell function from fasting plasma glucose and insulin concentrations in man. Diabetologia.

[19] Tang Q., Li X., Song P., Xu L. (2015). Optimal cut-off values for the homeostasis model assessment of insulin resistance (HOMA-IR) and pre-diabetes screening: Developments in research and prospects for the future. Drug Discov. Ther..

[20] Navarro-González D., Sánchez-Íñigo L., Pastrana-Delgado J., Fernández-Montero A., Martinez J.A. (2016). Triglyceride-glucose index (TyG index) in comparison with fasting plasma glucose improved diabetes prediction in patients with normal fasting glucose: The Vascular-Metabolic CUN cohort. Prev. Med..

[21] Ramos-Lopez O., Riezu-Boj J.I., Milagro F.I., Cuervo M., Goni L., Martinez J.A. (2019). Models Integrating Genetic and Lifestyle Interactions on Two Adiposity Phenotypes for Personalized Prescription of Energy-Restricted Diets With Different Macronutrient Distribution. Front. Genet..

[22] Excoffier L., Laval G., Schneider S. (2007). Arlequin (version 3.0): An integrated software package for population genetics data analysis. Evol. Bioinform. Online.

[23] Van der Aa M.P., Fazeli Farsani S., Knibbe C.A., de Boer A., van der Vorst M.M. (2015). Population-Based Studies on the Epidemiology of Insulin Resistance in Children. J. Diabetes Res..

[24] Ziaee A., Esmailzadehha N., Oveisi S., Ghorbani A., Ghanei L. (2015). The threshold value of homeostasis model assessment for insulin resistance in Qazvin Metabolic Diseases Study (QMDS): Assessment of metabolic syndrome. J. Res. Health Sci..

[25] Timóteo A.T., Miranda F., Carmo M.M., Ferreira R.C. (2014). Optimal cut-off value for homeostasis model assessment (HOMA) index of insulin-resistance in a population of patients admitted electively in a Portuguese cardiology ward. Acta Medica Port..

[26] Ye J. (2013). Mechanisms of insulin resistance in obesity. Front. Med..

[27] Mahalle N., Kulkarni M.V., Naik S.S., Garg M.K. (2014). Association of dietary factors with insulin resistance and inflammatory markers in subjects with diabetes mellitus and coronary artery disease in Indian population. J. Diabetes Complicat..

[28] White J., Jago R., Thompson J.L. (2014). Dietary risk factors for the development of insulin resistance in adolescent girls: A 3-year prospective study. Public Health Nutr..

[29] Macdonald I.A. (2016). A review of recent evidence relating to sugars, insulin resistance and diabetes. Eur. J. Nutr..

[30] Lau C., Faerch K., Glümer C., Tetens I., Pedersen O., Carstensen B., Jørgensen T., Borch-Johnsen K. (2005). Dietary glycemic index, glycemic load, fiber, simple sugars, and insulin resistance: The Inter99 study. Diabetes Care.

[31] Basciano H., Miller A.E., Naples M., Baker C., Kohen R., Xu E., Su Q., Allister E.M., Wheeler M.B., Adeli K. (2009). Metabolic effects of dietary cholesterol in an animal model of insulin resistance and hepatic steatosis. Am. J. Physiol. Endocrinol. Metab..

[32] Fernandez L.M., Andersen C.J. (2014). Effects of dietary cholesterol in diabetes and cardiovascular disease. Clin. Lipidol..

[33] Weickert M.O., Pfeiffer A.F.H. (2018). Impact of Dietary Fiber Consumption on Insulin Resistance and the Prevention of Type 2 Diabetes. J. Nutr..

[34] Brown A.E., Walker M. (2016). Genetics of Insulin Resistance and the Metabolic Syndrome. Curr. Cardiol. Rep..

[35] Karaderi T., Drong A.W., Lindgren C.M. (2015). Insights into the Genetic Susceptibility to Type 2 Diabetes from Genome-Wide Association Studies of Obesity-Related Traits. Curr. Diabetes Rep..

[36] Ingelsson E., McCarthy M.I. (2018). Human Genetics of Obesity and Type 2 Diabetes Mellitus: Past, Present, and Future. Circ. Genom. Precis. Med..

[37] Xi B., Zhao X., Shen Y., Wu L., Hou D., Cheng H., Mi J. (2014). An obesity genetic risk score predicts risk of insulin resistance among Chinese children. Endocrine.

[38] Graae A.S., Hollensted M., Kloppenborg J.T., Mahendran Y., Schnurr T.M., Appel E.V.R., Rask J., Nielsen T.R.H., Johansen M.Ø., Linneberg A. (2018). An adult-based insulin resistance genetic risk score associates with insulin resistance, metabolic traits and altered fat distribution in Danish children and adolescents who are overweight or obese. Diabetologia.

[39] Ordovas J.M., Shen J. (2008). Gene-environment interactions and susceptibility to metabolic syndrome and other chronic diseases. J. Periodontol..

[40] Chen L., Chen R., Wang H., Liang F. (2015). Mechanisms Linking Inflammation to Insulin Resistance. Int. J. Endocrinol..

[41] Lihn A.S., Pedersen S.B., Richelsen B. (2005). Adiponectin: Action, regulation and association to insulin sensitivity. Obes. Rev..

[42] Hong K.W., Kim S.H., Zhang X., Park S. (2018). Interactions among the variants of insulin-related genes and nutrients increase the risk of type 2 diabetes. Nutr. Res..

[43] Kim D.S., Kim B.C., Daily J.W., Park S. (2018). High genetic risk scores for impaired insulin secretory capacity doubles the risk for type 2 diabetes in Asians and is exacerbated by Western-type diets. Diabetes Metab. Res. Rev..

[44] Daily J.W., Liu M., Park S. (2019). High genetic risk scores of SLIT3, PLEKHA5 and PPP2R2C variants increased insulin resistance and interacted with coffee and caffeine consumption in middle-aged adults. Nutr. Metab. Cardiovasc. Dis..

[45] Cuevas-Sierra A., Ramos-Lopez O., Riezu-Boj J.I., Milagro F.I., Martinez J.A. (2019). Diet, Gut Microbiota, and Obesity: Links with Host Genetics and Epigenetics and Potential Applications. Adv. Nutr..

[46] Ramos-Lopez O., Cuervo M., Goni L., Milagro F.I., Riezu-Boj J.I., Martinez J.A. (2019). Modeling of an integrative prototype based on genetic, phenotypic and environmental information for personalized prescription of energy-restricted diets in overweight/obese subjects. Am. J. Clin. Nutr..

