# Supplementary material for: Interplay of an Obesity-Based Genetic Risk Score with Dietary and Endocrine Factors on Insulin Resistance
Source: Nutrients. 2019 Dec 21;12(1):33. doi: 10.3390/nu12010033 (PMC7019905; doi:10.3390/nu12010033)
Supplement: Supplementary file 1 [file nutrients-12-00033-s001.pdf]

Table S1. Anthropometric, clinical and biochemical characteristics of the study population categorized by insulin resistance genetic risk categories.

Variable	LGRS	HGRS	P value
Age (y)	46.6±0.4	45.6±0.2	0.456
Sex (F/M)	86/30	75/41	0.088
Anthropometrics and clinical data			
Weight (kg)	84.6±0.6	89.9±0.8	**0.003**
BMI (kg/m^2)	30.9±0.4	32.3±0.2	**0.004**
WC (cm)	101.1±0.4	102.7±0.5	**0.020**
TFAT (kg)	36.6±0.04	36.9±0.4	0.586
VFAT (kg)	1.37±0.04	1.48±0.05	0.052
SBP (mmHg)	129±1	128±2	0.639
DBP (mmHg)	78±1	81±1	0.137
Biochemical profile			
Glucose (mg/dL)	94.7±0.9	96.6±1.0	0.163
Insulin (mU/L)	6.4±0.4	9.2±0.4	**<0.001**
HOMA-IR index	1.53±0.11	2.28±0.11	**<0.001**
Total cholesterol (mg/dL)	218.7±3.6	215.8±3.9	0.572
LDL-c (mg/dL)	143.0±3.2	139.4±3.2	0.441
HDL-c (mg/dL)	57.1±1.1	55.0±1.5	0.213
Triglycerides (mg/dL)	93.2±4.7	106.6±4.8	**0.049**
TyG index (ratio)	8.29±0.04	8.43±0.04	**0.022**
Uric acid (mg/dL)	5.13±0.10	5.12±0.11	0.926
ALT (IU/L)	20.7±1.4	26.8±1.4	**0.002**
AST (IU/L)	20.8±0.9	23.4±0.9	0.051
Adiponectin (μg/mL)	11.9±0.4	11.0±0.4	0.143
Leptin (ng/mL)	36.6±2.1	38.0±2.0	0.644
CRP (μg/mL)	2.50±0.25	2.92±0.26	0.250
TNFα (pg/mL)	1.01±0.04	0.90±0.03	0.769

Variables are expressed as means ± standard errors. LGRS: low genetic risk score; HGRS: high-genetic risk score; BMI: body mass index; WC: waist circumference; TFAT: total body fat; VFAT: visceral fat; SBP: systolic blood pressure; DBP: diastolic blood pressure; LDL-c: low-density lipoprotein cholesterol; HDL-c: high-density lipoprotein cholesterol; ALT: alanine aminotransferase; AST: aspartate aminotransferase; CRP: C-reactive protein; TNFα: tumoral necrosis factor alpha; TyG index: triglyceride-glucose index. HOMA-IR index: Homeostatic Model Assessment Insulin Resistance index. Comparisons were performed by ANCOVA tests adjusted by age, sex, and BMI. Bold numbers indicate $P < 0.05$. NIR: HOMA-IR index ≤ 2.5; IR: HOMA-IR index > 2.5.

Table S2. Nutritional profile and physical activity patterns of the study subjects according to insulin resistance genetic risk categories.

Variable	LGRS	HGRS	P value
Energy (kilocalories/d)	1976±48	1957±49	0.789
Macronutrients			
Complex carbohydrates (%E/d)	23.1±0.6	23.4±0.6	0.709
Simple carbohydrates (%E/d)	19.5±0.5	19.0±0.6	0.524
Total protein (%E/d)	19.2±0.4	20.0±0.4	0.182
Animal protein (%E/d)	13.3±0.4	13.8±0.4	0.384
Vegetal protein (%E/d)	5.4±0.2	5.5±0.2	0.689
Total fat (%E/d)	37.5±0.7	37.1±0.7	0.608
SFA (%E/d)	10.3±0.3	10.3±0.4	0.942
MUFA (%E/d)	16.0±0.3	15.7±0.3	0.635
PUFA (%E/d)	4.8±0.1	4.8±0.2	0.984
Cholesterol (mg/d)	385±18	407±18	0.414
Fiber (g/d)	21.8±0.7	21.3±0.7	0.624
Water (ml/d)	1139±30	1139±31	0.997
Lifestyle			
Physical activity (METs/d)	23.7±1.8	22.7±1.8	0.681

Variables are expressed as means ± standard deviations. Average intakes of macronutrients are adjusted by total energy consumption. LGRS: low genetic risk score; HGRS: high-genetic risk score; SFA: saturated fatty acids; MUFA: monounsaturated fatty acids; PUFA: polyunsaturated fatty acids; METs: metabolic equivalents. Comparisons were performed by ANCOVA tests adjusted by age, sex, and BMI.

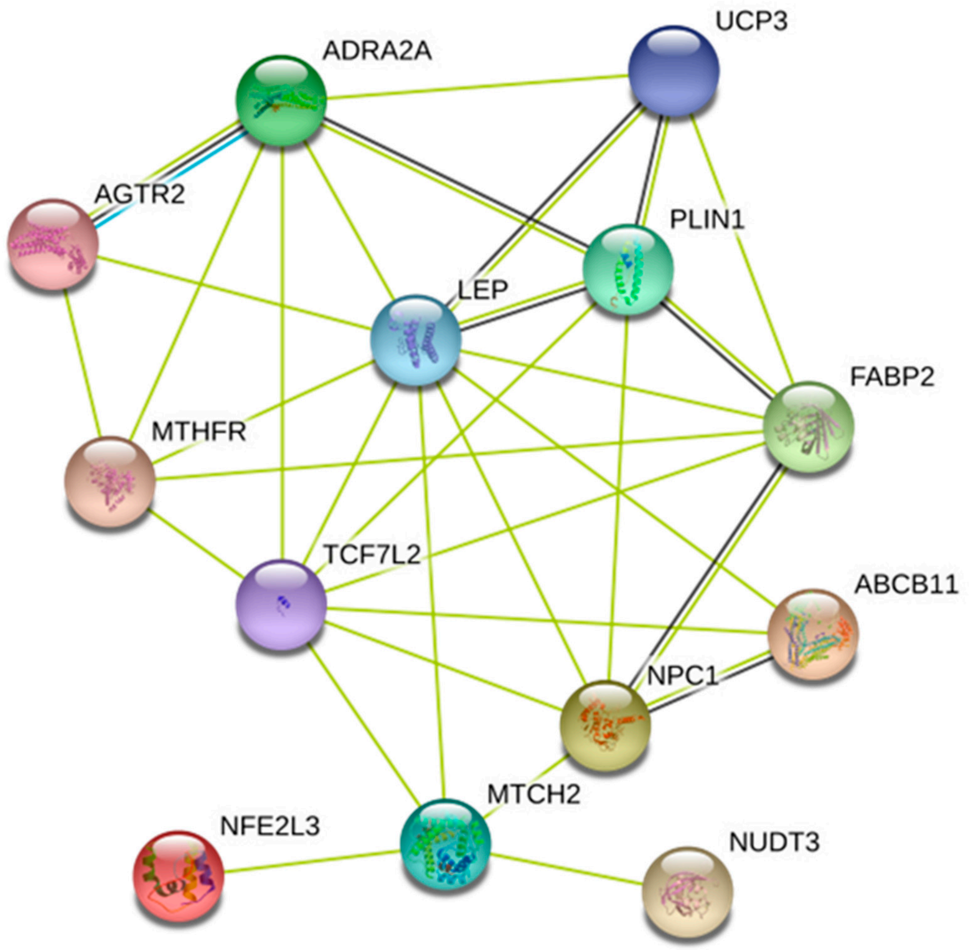

Figure S1. Multiprotein network showing potential interactions between the 13 genes where SNPs are present. PPI enrichment p-value of 5.71e-14.
